# Mechanical and Metallurgical Characterization of Nickel-Titanium Wire Types for Rotary Endodontic Instrument Manufacture

**DOI:** 10.3390/ma15238367

**Published:** 2022-11-24

**Authors:** Philip Y-H. Chien, Jorge N. R. Martins, Laurence J. Walsh, Ove A. Peters

**Affiliations:** 1 School of Dentistry, The University of Queensland, Brisbane, QLD 4006, Australia; 2 Faculdade de Medicina Dentária, Universidade de Lisboa, 1600-277 Lisboa, Portugal; 3 Grupo de Investigação em Bioquimica e Biologia Oral, Unidade de Investigação em Ciências Orais e Biomédicas (UICOB), 1600-277 Lisboa, Portugal; 4 Centro de Estudo de Medicina Dentária Baseada na Evidência (CEMDBE), 1600-277 Lisboa, Portugal

**Keywords:** austenite, dental instrument, differential scanning calorimetry, endodontics, martensite, nickel-titanium, R-phase, strain, stress, tensile test

## Abstract

This study aimed to evaluate and compare the effects of ambient temperature and post-manufacture heat-treatment on the mechanical behavior of nickel-titanium (NiTi) wires. Four types of commercial NiTi variants (Stock NiTi, heat treated “Blue”, “Gold”, “Superflex”, all Dentsply Maillefer, Ballaigues, Switzerland) were stressed in a tensile testing machine in a temperature-controlled water bath at three different temperatures. Stress and strain values were extrapolated from the raw data, and 2-way ANOVA and Tukey’s test for multiple comparisons were performed to compare the differences of the mechanical constants. Differential scanning calorimetry (DSC) tests established the martensitic transformation starting (M_s_), finishing (M_f_) and austenitic (reverse-martensitic) starting (A_s_) and finishing (A_f_) points. Austenitic modulus of elasticity and transformation stress values increased with temperature for all NiTi groups. The martensitic modulus of elasticity, maximum transformation strain and ultimate tensile stress were not significantly affected by temperature changes. Stock NiTi and Gold wire samples presented with clearly delineated austenitic and martensitic transformations in the DSC experiments. Differences in manufacturing/heat treatment conditions and ambient temperature affect the mechanical behavior of nickel-titanium and may have clinical implications. Further improvements to the experimental setup could be considered to provide more accurate measurements of strain.

## 1. Introduction

Nickel-titanium (NiTi) alloys have been employed in the production of handheld endodontic instruments since 1988 by machining orthodontic wire. The production of rotary endodontic instruments from NiTi wire blanks is still the primary method to this day [1,2]. In brief, NiTi rotary instruments are produced by vacuum melting or casting of equiatomic NiTi alloy (56% (weight [wt]) nickel and 44% (wt) titanium), which is then press-forged into a cylindrical shape, followed by a complex sequence of swaging, rolling, drawing, descaling, annealing and profiling the wire into its desired shape [1]. In addition to innovations in instrument design and morphology, several proprietary processing procedures for NiTi rotary endodontic instruments have been developed over the last decade to improve their mechanical properties [3].

**Figure 1:**
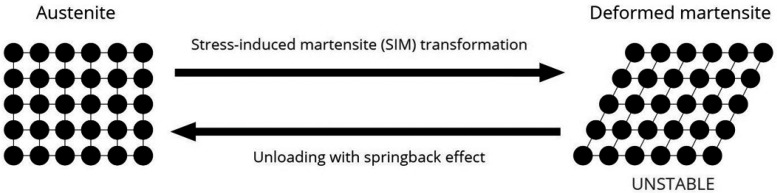
Demonstration of superelasticity (SE) through stress-induced martensite transformation (SIM) within 8% elastic deformation. The martensite is in an unstable monoclinic lattice structure and returns to its austenitic state upon the unloading of stress.

**Figure 2:**
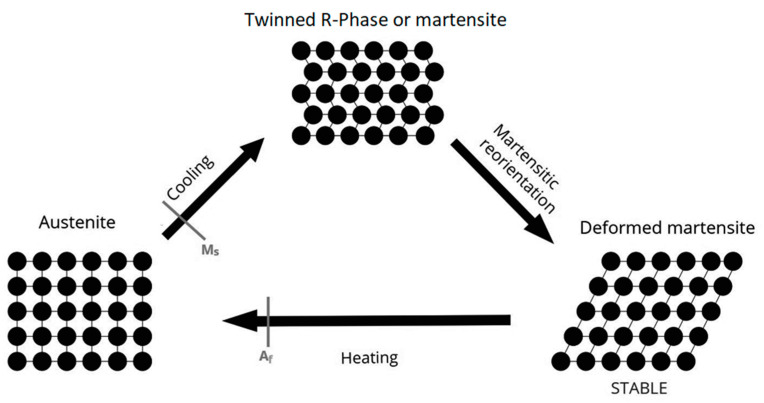
Demonstration of shape memory effect (SME). Austenite (above the austenite finish temperature, A_f_) is cooled to a twinned martensitic phase (from the martensitic start temperature, M_s_) known as R-phase. As NiTi in the R-phase cannot be ground, it is instead twisted and reoriented into a stable martensitic phase and is heated and cooled back into the austenite crystalline structure [6].

The two temperature-dependent crystal structures in which near to equiatomic NiTi alloy can exist are austenite (high temperature phase with cubic B2 crystal structure) and martensite (low-temperature with monoclinic B19′ crystal structure) [3,4]. Both stress and temperature can induce the austenite-to-martensite transition (martensitic transformation), and it is this transition that gives NiTi its characteristic properties of superelasticity (SE) and shape memory effect (SME) [3]. SE is the complete recoverable elastic deformation (within 8% strain limit) due to the phase transformation between stable austenite and the stress-induced martensite phase, whereas SME is the ability of deformed NiTi to revert to its original shape as a result of phase transformation from heating stable deformed martensite to austenite [1,3]. Under certain conditions, a “pre-martensitic transition” with a rhombohedrally distorted phase (R-phase) can precede the martensitic transformation [5], and one of these conditions is met via a process of heating and cooling NiTi in their austenitic state [6], as R-phase transformations tend to occur when precipitates or dislocations exist. [7] Transformations between cubic, rhombohedral and monoclinic lattice structures are summarized in Figure 1 and Figure 2.

If a NiTi alloy is above the austenitic finish temperature (A_f_), it is considered austenitic, which is stiff, hard and possesses superior SE properties [3,7]. If the temperature is below the martensitic finish temperature (M_f_), the NiTi alloy is soft, ductile and possesses SME [3,7]. Given the ability for martensite to reorient itself in its twinned R-phase structure, martensite exhibits superior cyclic fatigue resistance [3,4,8], and is therefore considered more desirable when the preparation of curved root canals is required.

The present study hypothesized that different types of post-manufacture heat treatment would affect material constants in a specific pattern and align with changes in transformation temperatures. Microtensile tests and differential scanning calorimetry (DSC) were employed to study the effects of thermal processes on the mechanical and metallurgical properties of NiTi alloys.

## 2. Materials and Methods

**Figure 3:**
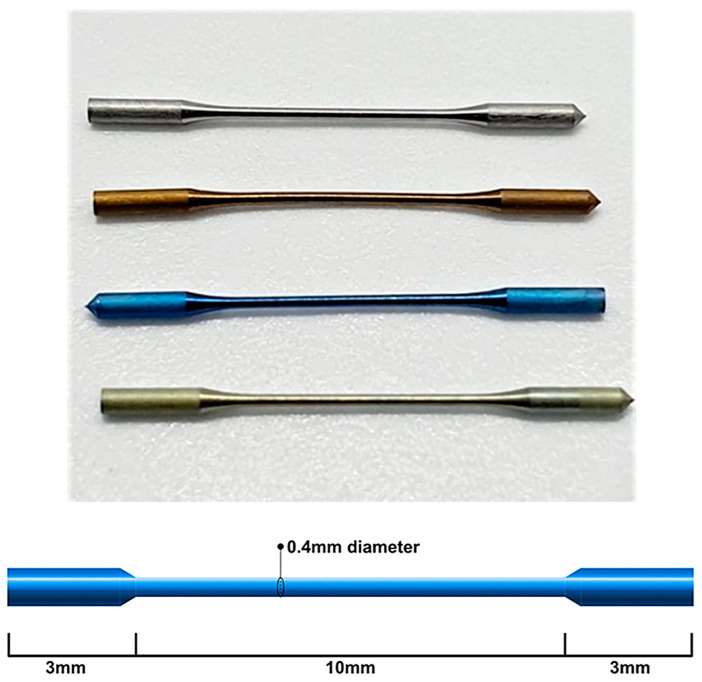
Wire blank specimens purpose-made for the fabrication of Dentsply endodontic instruments. Top: Photograph of Stock, Gold, Blue and Superflex wire blanks. Bottom: Schematic diagram showing the dimensions of each wire blank.

Four different types of NiTi wire specimens were sourced from Dentsply Maillefer (Ballaigues, Switzerland). All samples were manufactured to a 0.4 mm cross-sectional diameter and a total length of 16 mm, of which the central 10 mm portion was sized to the relevant gauge; samples for three groups had then been subjected to typical post-manufacture heat treatment. The shape and dimensions of the wires were based on materials described in a previous study [9]. The wire type and the corresponding proprietary endodontic instrument brands are detailed in Table 1, while the dimensions are detailed in Figure 3. The ends of the wire samples had been milled to a larger diameter to facilitate the grip mechanism for the uniaxial test machine. Of note, “Superflex” is an undisclosed proprietary NiTi type that has been used in the fabrication of the TruNatomy^TM^ (Dentsply Sirona, Charlotte, NC, USA) instrument.

### 2.1. Microtensile Testing

**Figure 4:**
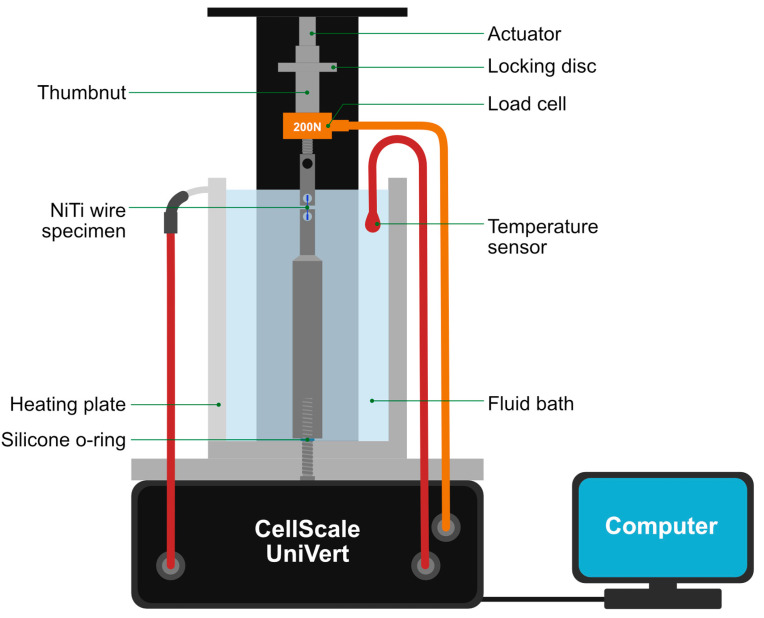
Schematic of UniVert microtensile tester.

**Figure 5:**
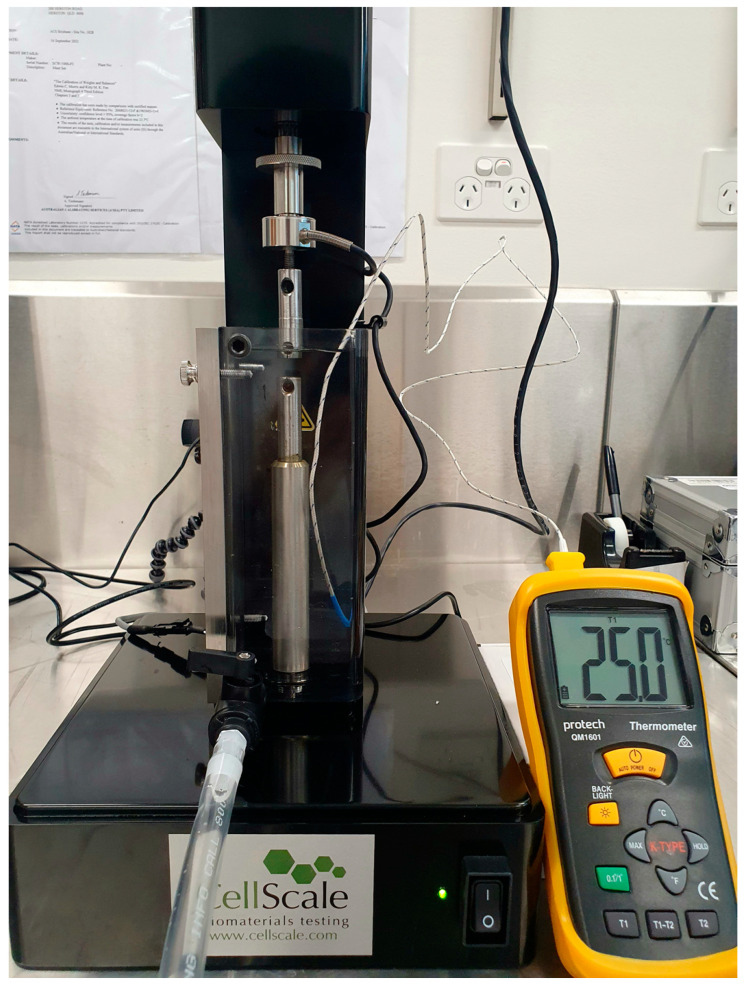
Photograph of UniVert microtensile test setup used.

Two custom-made stainless steel grips were used in a microtensile test rig (UniVert, CellScale, Waterloo, ON, Canada) to attach the wire specimens. Stainless steel tubes were milled to fit the actuator and base components of the UniVert machine and aligned rigidly in the same axis of the actuator. The tester was fitted with a water bath, a heating plate and a temperature sensor to provide a pre-selected ambient temperature (Figure 4 and Figure 5). An externally placed thermocouple with an accuracy of ±0.1 °C was used to monitor the temperature of the water bath and to ensure that this was maintained at ±1.0 °C.

Three samples of each wire type (Stock, Gold, Blue, Superflex) were tested at one of three temperatures: cold (12.5 °C), room temperature (25 °C), and body temperature (37.5 °C). A 200 N load cell at an extension rate of 0.1 mm per minute was selected as detailed in ASTM E8/E8M-21 [10]. Each sample was examined at 25× magnification to ensure that no pre-existing defects were present. Stress values were derived from the output from the load cell (in Newtons) and converted to stress σ in megaPascals (MPa) by using the formula in Equation (1).

(1) $$\sigma =\frac{F}{A}$$

where *F* is the load recorded by the load cell, and *A* is the circular cross-sectional area of the gauge length.

**Figure 6:**
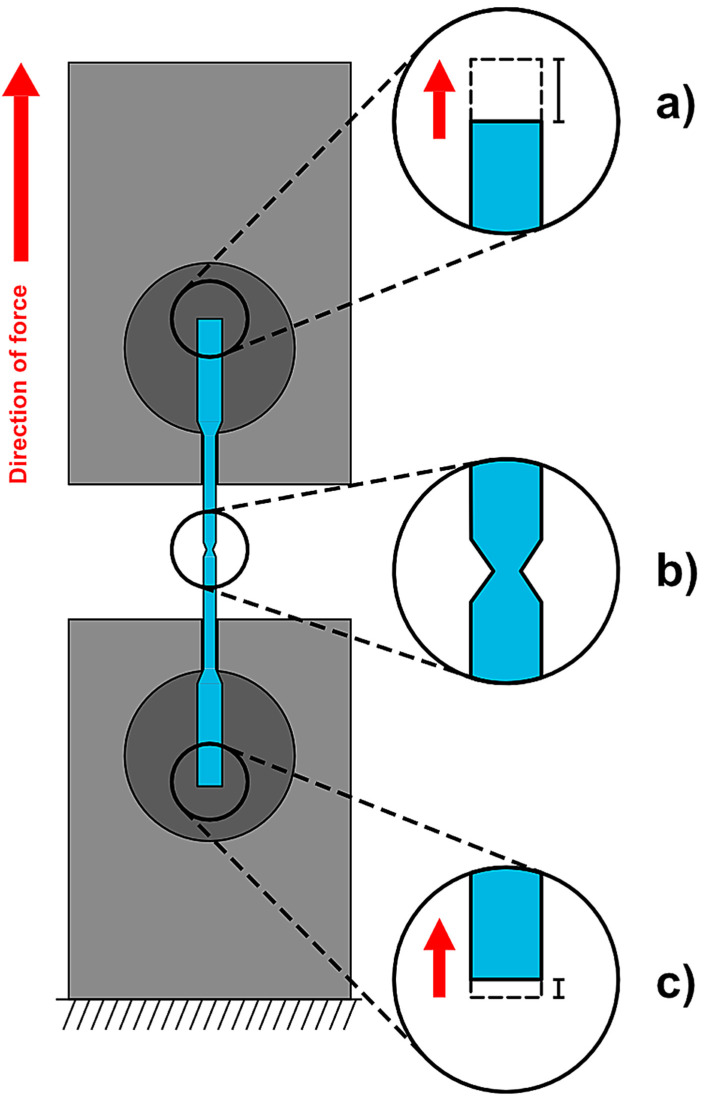
Schematic diagram of microtensile testing. (**a**) shows the upper part of the NiTi wire specimen during loading. The dotted portion indicates the final position of the specimen prior to failure. (**b**) shows a NiTi wire specimen in the frame immediately prior to ultimate tensile failure where the so-called necking effect is visible in the gauge portion of the wire. (**c**) shows the bottom part of the NiTi wire specimen during loading. The dotted portion indicates where the specimen initially began and moved vertically once the slack was removed from the grips. The difference in movement of the top and bottom of the parts of the specimen determine the true extensions, converted from pixels (px) to millimeters (mm).

A digital image correlation (DIC) technique was used to obtain strain measurements. Images were captured using a high-resolution webcam (Logitech HD Pro Webcam C920, Logitech, Lausanne, Switzerland) at a rate of 1 Hz, which corresponded to output data from the UniVert (Figure 6). The extensions until ultimate tensile failure were analyzed using ImageJ software using the difference in pixels, converted to measurements in millimeters (mm) by calibration of images using fixed landmarks with known dimensions in the images. Using the formula in Equation (2), strain values were calculated.

(2) $$\varepsilon =\frac{∆L}{L_{0}}$$

where ∆*L* is the change in length and *L*_0_ is the original gauge length of the wire (10 mm).

The stress and strain values were plotted and analyzed for austenitic and martensitic modulus of elasticity (E_a_ and E_m_, respectively). Values for maximum transformation strain (%), transformation stress [σ_A-M_] (MPa) and ultimate tensile strength [σ_max_] (MPa) were also derived.

Data sets were first checked for normality of distribution via the Shapiro–Wilk test, and subsequently 2-way ANOVA and Tukey’s post hoc test for multiple comparisons were used (Prism version 9, GraphPad Software, San Diego, CA, USA). Two separate one-way ANOVA tests were performed to analyze the effect of temperature within each NiTi group, and the effect of the metallurgical types of NiTi at similar temperatures. The threshold for statistical significance was *p* < 0.05.

The following null hypotheses were proposed:

- Mean values of the mechanical properties are not statistically significant for identical NiTi types at different temperatures.
- Mean values of the mechanical properties are not statistically significant for different NiTi types at identical temperatures.

### 2.2. Differential Scanning Calorimetry (DSC)

Three samples from each of the four wire types were used for DSC analysis. Sections of 3 mm in length were cut carefully off and weighed to an accuracy of ±0.001 mg before being placed in a standardized 40 µL aluminum crucible (Mettler Toledo, Greifensee, Switzerland), and sealed non-hermetically with the Mettler Toledo crucible sealing press. The samples were prepared so that they had a mass of between 10–12 mg, and as such the thickened end of the wires was used for each specimen to meet the weight requirements for DSC. An empty aluminum crucible was used as the reference pan.

Each sample was placed in the DSC unit (DSC 1 STARe system, Mettler Toledo) along with the reference pan. The temperature range varied from −60 °C to +60 °C. Liquid nitrogen was used to achieve sub-ambient temperature conditions. The heating and cooling cycles followed the protocol described in de Vasconcelos et al., 2016 [11]. The samples were first heated to +60 °C, then cooled to −60 °C at a rate of −10 °C/min, and then immediately heated back to +60 °C at a rate of +10 °C/min, with one cycle per sample.

All DSC data were analyzed using STARe Evaluation software (Mettler Toledo). The onset and endset transformation temperatures were obtained from the intersection between extrapolations of the baseline and maximum gradient line of the lambda-type DSC curve [12,13], with four main points determined: martensitic transformation starting (M_s_), finishing (M_f_) and austenitic (reverse-martensitic) starting (A_s_) and finishing (A_f_).

## 3. Results

### 3.1. Microtensile Testing

**Figure 7:**
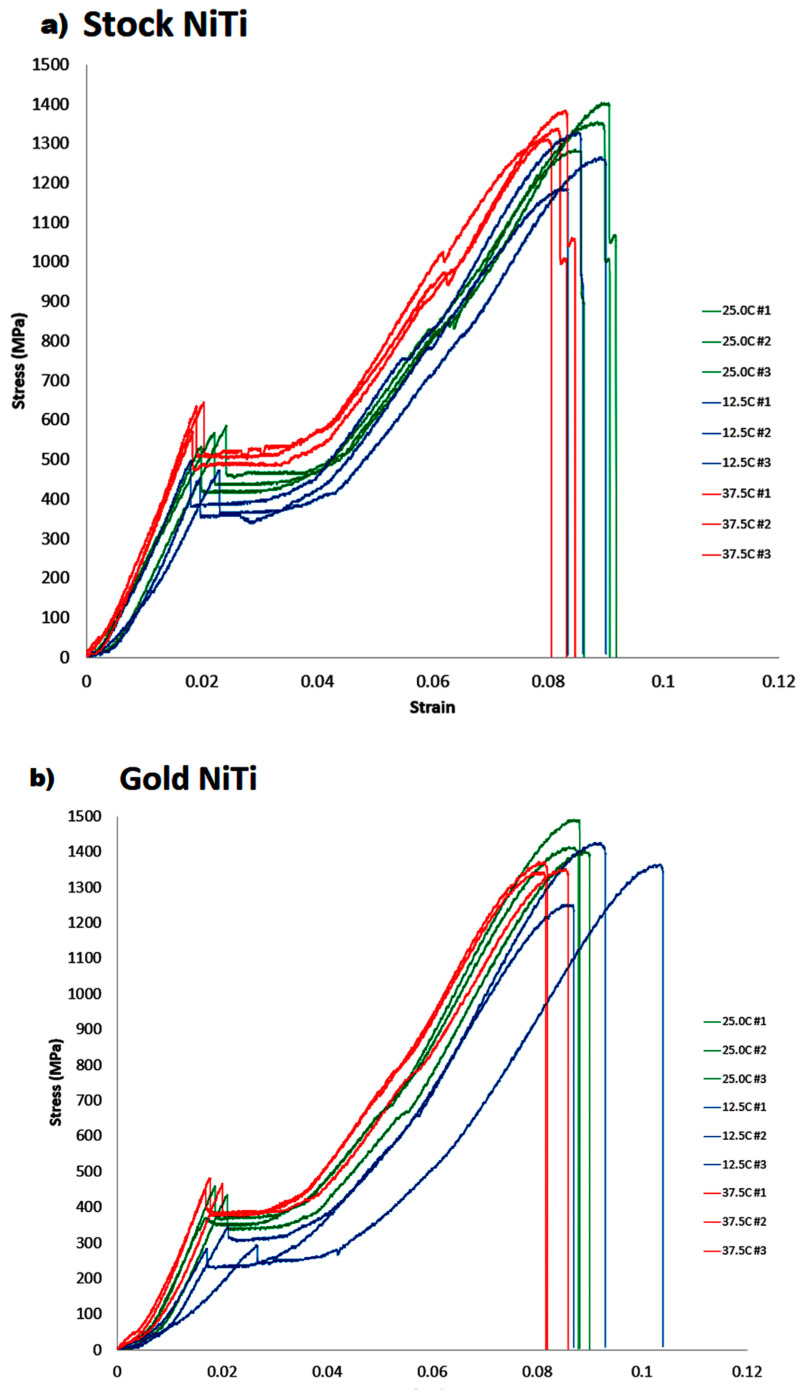

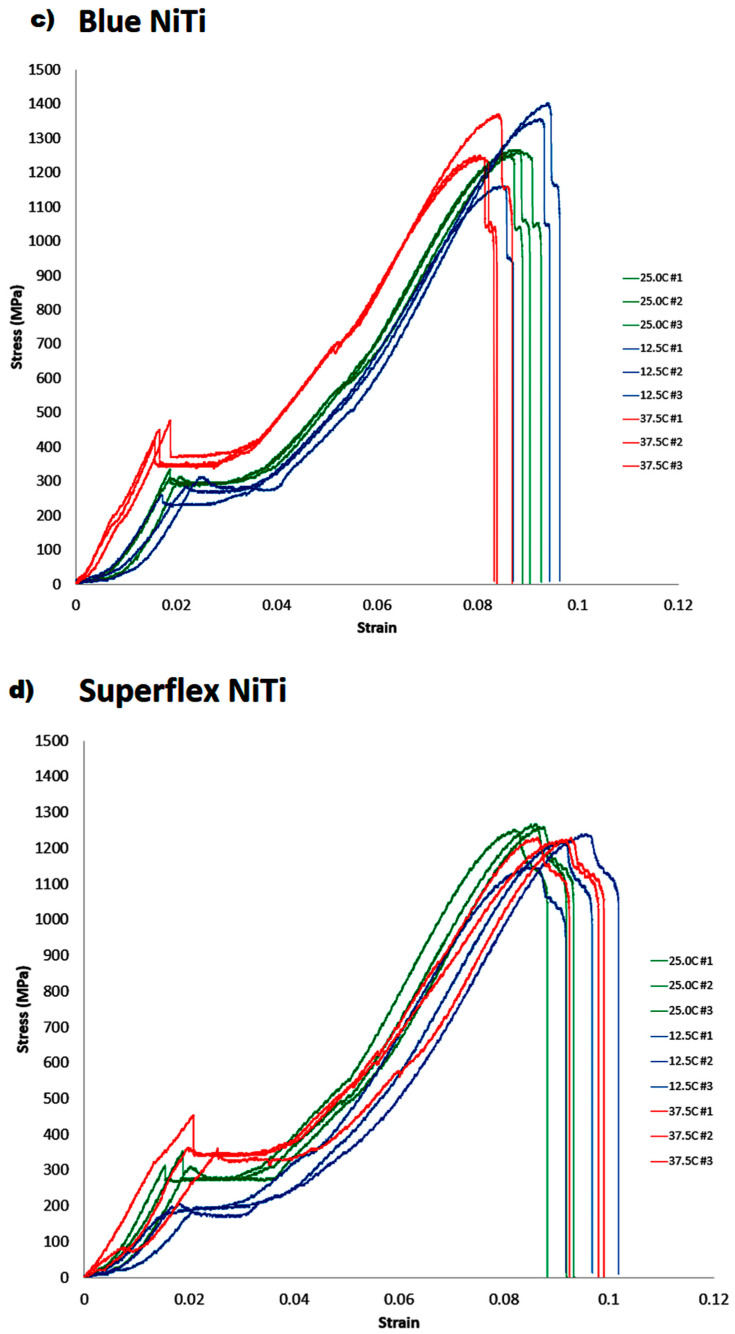
Stress–strain graphs of Stock (**a**), Gold (**b**), Blue (**c**) and Superflex (**d**) NiTi conducted across 12.5 °C, 25.0 °C, and 37.5 °C.

Microtensile test results were consistent within each sample group (Table 2). In general, Ea and σ_A-M_ values increased with temperature for all four groups of NiTi wire samples, while E_m_, ε_max_ and σ_max_ did not appear to be affected by temperature changes (Table 3). At 37.5 °C, all four NiTi types were significantly different from each other for all mechanical properties, but at 12.5 °C only E_a_ and σ_A-M_ were significantly different. At 25 °C only ε_max_, σ_A-M_ and σ_max_ were significantly different (Table 4). The stress–strain graphs of all conducted experimental runs are shown in Figure 7.

### 3.2. DSC Analysis

The four wire types had different transformation temperatures (Table 5). Stock and Gold wires samples showed clear austenitic and martensitic transformations, whereas Blue and Superflex wire samples included some intermediate phases, suggestive of R-phase transition phases. The Blue wire showed a small transition peak at around 21.3 °C, with a major peak at 31.8 °C.

**Figure 8:**
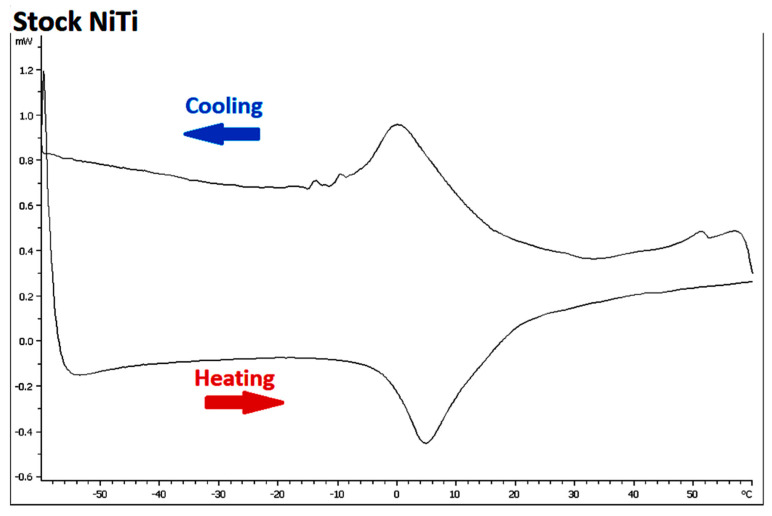

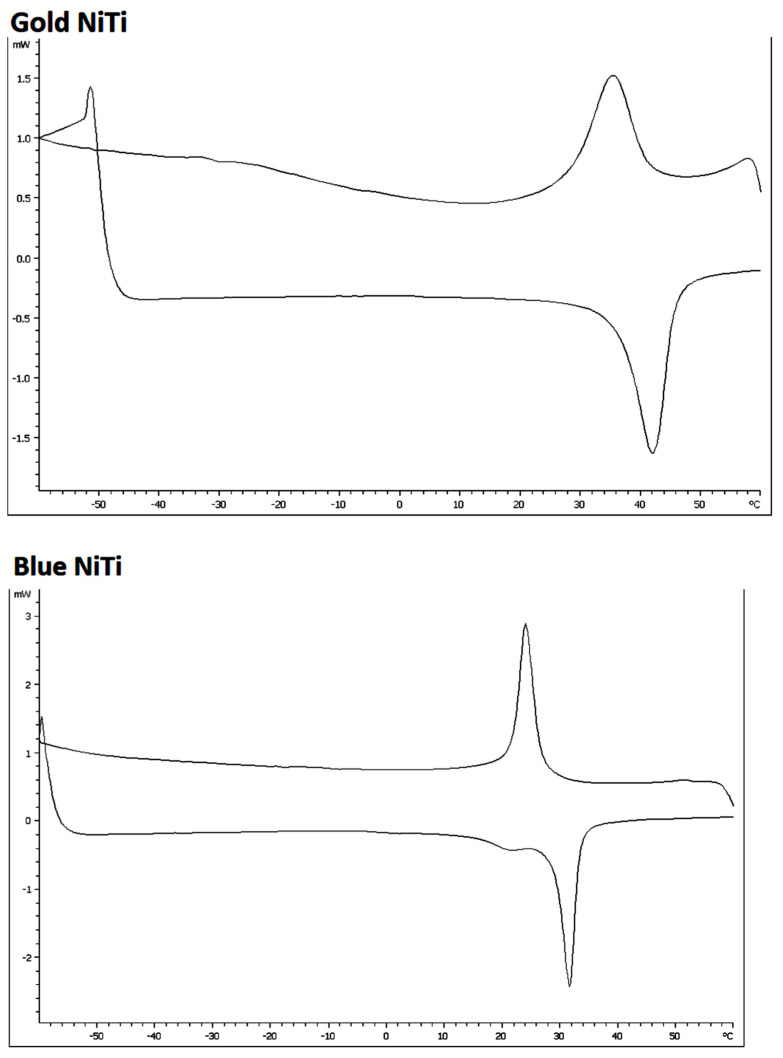

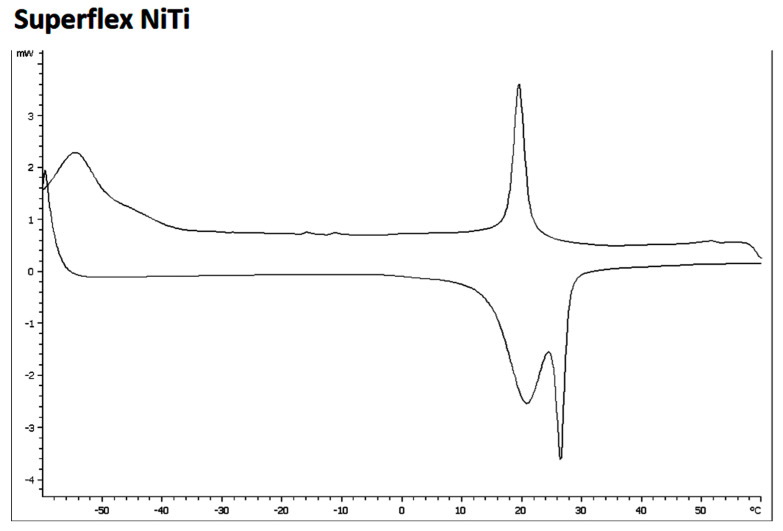
DSC curves for Stock, Gold, Blue and Superflex wires, indicating heat flow (mW) during cooling (upper trace) and heating (lower trace).

The Stock NiTi alloy displayed the expected austenite-martensite phase transformation centered close to 0 °C (Figure 8). Gold, Blue and Superflex samples all showed much higher temperature austenitic and martensitic transformations than the Stock NiTi alloy. The Blue wire showed a small transition peak in the reverse martensitic phase at around 21.3 °C, with its major peak at 31.8 °C. The Superflex wire presented two very pronounced peaks in the reverse martensitic phase, with the first peak at 20.9 °C and the second peak at 26.9 °C.

## 4. Discussion

The results from this study show that heat-treated NiTi samples have different mechanical properties at cold, room and body temperatures compared to the untreated SE (Stock) control, with the latter two being relevant to clinical practice in endodontics. This is important given that some past studies of NiTi instruments have been criticized for limited relevance to the clinical setting [14]. A research approach incorporating mechanical testing and metallurgical characterization has been adopted in more recent studies [15]. The present study is the first to use a temperature-controlled water bath to explore the effects of temperature on the mechanical properties of samples of differing NiTi alloy types. Water was the selected medium over other possibilities such as isotonic aqueous sodium chloride solution to prevent the corrosion of the apparatus, however other fluid mediums that more closely resemble biological fluids could be explored in future.

The present study demonstrates that both material type and ambient temperature influence the physical properties of NiTi alloys. The difference in sample color reflects variations in the thickness of the titanium oxide layer, which is a result of heat treatment [3]. In terms of the influence of temperature, values for ε_max_ and σ_max_ were not affected, while for all alloys, σ_A-M_ values were significantly higher at 37.5 °C than at 12.5 °C. This could have clinical relevance, as increased flexibility of instruments at body temperature should reduce the likelihood of instrument separation. At 37.5 °C, E_a_ was significantly lower for the Superflex alloy (24,395 ± 3896 MPa) when compared to Stock (35,155 ± 1498 MPa) and Gold alloys (33,061 ± 3597 MPa). This behavior could be caused by a larger initial R-phase in the Superflex alloy.

The DSC results of the present study are comparable to those reported in prior investigations for Stock [11,12,16,17], Gold [18,19] and Blue [11,16,20,21] NiTi alloys. The Stock NiTi alloy samples behave overall differently compared to the three heat-treated NiTi samples examined, with considerably lower transition temperatures for both the austenitic and martensitic phases. During the heating phase of Blue NiTi alloy, a small initial peak occurred, suggesting a transformation of from a martensitic to a rhombohedral intermediate “R-phase”, with a second more pronounced peak for the R-phase to austenite transformation [21]. The transition of martensite to R-phase to austenite is similar to that observed in the Superflex NiTi alloy, which performs similarly to Blue NiTi. The smaller peaks noted in the Stock NiTi alloy in the cooling phase are likely due to impurities in the reference pan, which is a well-documented source of error in DSC experiments [22,23]. Testing the samples with a larger temperature range (e.g., from −100 °C to +100 °C) could also confirm whether there were more transformation peaks in those temperature ranges. Future studies could use X-ray powder diffraction (XRD) to explore further the phases of NiTi present, particularly for Superflex NiTi.

Past studies of physical properties of NiTi alloys used in endodontic files have used three-point bending, rotating-bending and uniaxial loading/unloading tests to obtain stress–strain curves [24,25]. The present study used uniaxial tensile loading until failure, at three different temperatures, with a mechanical tester that included a temperature-controlled water bath. All samples showed an initial rise to the end of the austenitic phase, followed by a sharp drop in the load once σ_A-M_ was reached. The latter could reflect an “over-stress” event initiating the transformation of a microstructurally locked structure. After the initial linear rise to σ_A-M_, a plateau phase occurs as atoms reorientate and slip past each other, prior to a martensitic transformation.

Although some individual experiments showed a small plateau during martensitic transformation, the tensile data did not clearly show R-phase for Gold, Blue and Superflex alloys. In Gold NiTi, ultimate tensile failure occurred abruptly, with no visible necking effect, whereas Stock and Blue NiTi wires had short necking periods prior to tensile failure.

The Superflex alloy showed a unique transition prior to ultimate tensile failure, with a prolonged creep-like transition occurring prior to complete failure. The σ_A-M_ and σ_max_ values observed for the Stock NiTi alloy were similar to those reported in a previous study, [24] however E_a_ and E_m_ values were slightly lower than those from a past investigation [25].

When considering the effect of alloy types, values for Em were not statistically different between the four alloys, at any of the three temperatures used (12.5, 25, and 37.5 °C). This suggests that in the martensitic state, the orientation of atoms has reached a new stable state. On the other hand, Gold, Blue and Superflex NiTi alloys had significantly lower mean values for σ_A-M_ than the Stock alloy, suggesting a lower stress requirement for these three NiTi types to transform into the superelastic state.

The present study is subject to several limitations. The specimens did not conform to the standard “dogbone” shape, as they were cylindrical in nature. The change to the usual standardized specimen shape could explain the off-centered locations of tensile failure. The specimen design in this study were based on specimens described earlier [9], however a different specimen design may be considered in future to minimize the slippage of specimens. Specimens and experimental setups as detailed in ASTM F2516-22 [26] could be considered to conform to standardized testing methods of NiTi tension testing, although the standard does not propose a suitable method of temperature control. In terms of the experimental setup, tracking changes in the sample length can be problematic as most mechanical testers generate an output of displacement from movement of the crosshead, and overestimation of strain is likely [27]. Contact extensometers or strain sensors/gauges are used commonly to determine true strain, rather than DIC, as used in the present study [28]. The advantage of using DIC is that it can account for any slippage that occurs from the sample grips. Theoretically, in the setup used in the present study, there should be no axial movement of the specimen once the slack has been taken up. Nevertheless, there may have been minor slippage of the specimens if the gripped areas were able to slide out of the grips. Two key limitations of DIC are image resolution, and the requirement for the camera to be positioned properly to avoid parallax error. It is difficult to apply a strain gauge onto small specimens that are submerged in a fluid medium. There may be value in using DIC with 3D imaging systems that allow dynamic tracking of samples. Improvements to the grip mechanism, strain measurements and DIC techniques could be made in future work.

## 5. Conclusions

The present study examined the effect of temperature on tensile testing of various NiTi specimens by using a novel experimental setup with a temperature-controlled water bath.

- Behavior under tensile loading was influenced by NiTi alloy type and also by ambient temperature.
- Enhanced superelastic properties at body temperature could have relevance to improved clinical performance of newer NiTi alloy types.

## Figures and Tables

**Table 1:** List of NiTi wire types with their corresponding rotary instruments (all by Dentsply Maillefer, Ballaigues, Switzerland).

NiTi Type	Corresponding Dentsply Instrument
Stock	ProTaper Universal^TM^
Gold	ProTaper Gold, WaveOne Gold, ProTaper Ultimate^TM^
Blue	Vortex Blue, ProTaper Ultimate^TM^
Superflex	TruNatomy^TM^

**Table 2:** Tensile test results for different alloy types at 3 ambient temperatures (outcomes expressed in means ± standard deviation [SD] with n = 3).

NiTi	Temperature (°C)	E_a_ (MPa) ^1^	E_m_ (MPa) ^2^	ε_max_ (%) ^3^	σ_A-M_ (MPa) ^4^	σ_max_ (MPa) ^5^
Stock	12.5	27,672 ± 4155	19,731 ± 1048	4.2 ± 0.3	473.7 ± 22.1	1258.0 ± 72.2
	25	28,248 ± 1806	20,146 ± 470	4.5 ± 0.3	561.8 ± 25.8	1343.9 ± 60.9
	37.5	35,155 ± 1498	20,760 ± 505	4.3 ± 0.1	617.1 ± 40.5	1344.2 ± 37.1
Gold	12.5	21,135 ± 3461	21,657 ± 3107	4.0 ± 0.6	308.3 ± 34.9	1346.2 ± 87.4
	25	30,573 ± 1891	21,254 ± 444	3.8 ± 0.2	420.6 ± 47.8	1435.6 ± 47.2
	37.5	33,061± 3597	22,001 ± 766	3.8 ± 0.1	464.6 ± 16.9	1354.7 ± 15.5
Blue	12.5	21,645 ± 1757	21,154 ± 931	3.9 ± 0.2	279.0 ± 16.4	1291.4 ± 121.9
	25	28,673 ± 1444	20,900 ± 485	3.9 ± 0.2	314.1 ± 19.8	1238.0 ± 6.7
	37.5	29,880 ± 4932	19,988 ± 702	3.5 ± 0.2	448.2 ± 31.1	1286.2 ± 68.8
Superflex	12.5	18,527 ± 1751	20,376 ± 1577	3.5 ± 0.3	203.0 ± 0.9	1201.6 ± 46.4
	25	26,580 ± 1060	21,029 ± 594	3.6 ± 0.2	326.0 ± 23.8	1258.7 ± 6.7
	37.5	24,395 ± 3896	19,251 ± 1346	3.8 ± 0.3	388.0 ± 58.2	1223.5 ± 6.2

^1^ Austenite elasticity; ^2^ Martensite elasticity; ^3^ Maximum transformation strain; ^4^ Transformation stress; ^5^ Ultimate tensile stress.

**Table 3:** Multiple statistical comparisons for the influence of temperature. Data show comparisons of E_a_, E_m_, ε_max_, σ_A-M_, and σ_max_ for samples within their own alloy groups, at different temperatures. Significant outcomes are summarised as follows: * = *p* < 0.05; ** = *p* < 0.01; *** = *p* < 0.001; **** = *p* < 0.0001.

NiTi	Temperature (°C)	E_a_ (MPa) ^1^	E_m_ (MPa) ^2^	ε_max_ (%) ^3^	σ_A-M_ (MPa) ^4^	σ_max_ (MPa) ^5^
Stock	12.5 vs. 25.0	ns	ns	ns	*p* = 0.0066 **	ns
	12.5 vs. 37.5	*p* = 0.011 *	ns	ns	*p* < 0.0001 ****	ns
	25.0 vs. 37.5	*p* = 0.0193 *	ns	ns	ns	ns
Gold	12.5 vs. 25.0	*p* = 0.0015 **	ns	ns	*p* = 0.0007 ***	ns
	12.5 vs. 37.5	*p* = 0.0001 ***	ns	ns	*p* < 0.001 ****	ns
	25.0 vs. 37.5	ns	ns	ns	ns	ns
Blue	12.5 vs. 25.0	*p* = 0.0172 *	ns	ns	ns	ns
	12.5 vs. 37.5	*p* = 0.0051 **	ns	ns	*p* < 0.0001 ****	ns
	25.0 vs. 37.5	ns	ns	ns	*p* < 0.0001 ****	ns
Superflex	12.5 vs. 25.0	*p* = 0.0062 **	ns	ns	*p* = 0.0002	ns
	12.5 vs. 37.5	ns	ns	ns	*p* < 0.0001 ****	ns
	25.0 vs. 37.5	ns	ns	ns	ns	ns

^1^ Austenite elasticity; ^2^ Martensite elasticity; ^3^ Maximum transformation strain; ^4^ Transformation stress; ^5^ Ultimate tensile stress.

**Table 4:** Multiple statistical comparisons for the influence of sample type. This table shows results for Tukey’s multiple comparisons comparing E_a_, E_m_, ε_max_, σ_A-M_, and σ_max_ at the same temperature for different alloys. Significant outcomes are summarised as follows: * = *p* < 0.05; ** = *p* < 0.01; *** = *p* < 0.001; **** = *p* < 0.0001.

Temperature (°C)	NiTi	E_a_ (MPa) ^1^	E_m_ (MPa) ^2^	ε_max_ (%) ^3^	σ_A-M_ (MPa) ^4^	σ_max_ (MPa) ^5^
12.5	Stock vs. Gold	*p* = 0.0485 *	ns	ns	*p* < 0.0001 ****	ns
	Stock vs. Blue	ns	ns	ns	*p* < 0.0001 ****	ns
	Stock vs. Superflex	*p* = 0.0037 **	n	*p* = 0.0270 *	*p* < 0.0001 ****	ns
	Gold vs. Blue	ns	ns	ns	ns	ns
	Gold vs. Superflex	ns	ns	ns	*p* = 0.0024 **	*p* = 0.0308 *
	Blue vs. Superflex	ns	ns	ns	*p* = 0.00350 *	ns
25	Stock vs. Gold	ns	ns	*p* = 0.0261 *	*p* < 0.0001 ****	ns
	Stock vs. Blue	ns	ns	ns	*p* < 0.0001 ****	ns
	Stock vs. Superflex	ns	ns	*p* = 0.0030 *	*p* < 0.0001 ****	ns
	Gold vs. Blue	ns	ns	ns	*p* = 0.0022 **	*p* = 0.0063 **
	Gold vs. Superflex	ns	ns	ns	*p* = 0.0067 **	*p* = 0.0065 **
	Blue vs. Superflex	ns	ns	ns	ns	ns
37.5	Stock vs. Gold	ns	ns	ns	*p* < 0.0001 ****	ns
	Stock vs. Blue	ns	ns	*p* = 0.0064 **	*p* < 0.0001 ****	ns
	Stock vs. Superflex	*p* = 0.0007 ***	ns	ns	*p* < 0.0001 ****	ns
	Gold vs. Blue	ns	ns	ns	ns	ns
	Gold vs. Superflex	*p* = 0.0061 **	ns	ns	*p* = 0.0332	ns
	Blue vs. Superflex	ns	ns	ns	ns	ns

^1^ Austenite elasticity; ^2^ Martensite elasticity; ^3^ Maximum transformation strain; ^4^ Transformation stress; ^5^ Ultimate tensile stress.

**Table 5:** Transformation temperatures for NiTi wire samples. Data show means ± standard deviation for N = 3 samples for each wire.

NiTi Type	M_s_ (°C) ^1^	M_f_ (°C) ^2^	A_s_ (°C) ^3^	A_f_ (°C) ^4^
Stock	13.0 ± 0.2	−7.6 ± 1.8	−3.0 ± 0.2	15.9 ± 0.5
Gold	41.4 ± 0.1	29.8 ± 1.2	36.3 ± 0.2	46.0 ± 0.2
Blue	27.2 ± 0.4	23.2 ± 1.5	29.3 ± 0.4	33.4 ± 0.1
Superflex	20.9 ± 1.1	17.5 ± 0.2	24.3 ± 0.1	28.2 ± 0.2

^1^ Martensite start temperature; ^2^ Martensite finish temperature; ^3^ Austenite start temperature; ^4^ Austenite finish temperature.
